# Expression of innate immunity genes in human hematopoietic stem/progenitor cells – single cell RNA-seq analysis

**DOI:** 10.3389/fimmu.2025.1515856

**Published:** 2025-04-08

**Authors:** Justyna Jarczak, Kannathasan Thetchinamoorthy, Diana Wierzbicka, Kamila Bujko, Mariusz Z. Ratajczak, Magdalena Kucia

**Affiliations:** ^1^ Laboratory of Regenerative Medicine, Medical University of Warsaw, Warsaw, Poland; ^2^ Stem Cell Institute at James Graham Brown Cancer Center, University of Louisville, Louisville, KY, United States

**Keywords:** innate immunity, complement cascade, toll-like receptors, NOD-like receptors, NLRP3 inflammasome, complosome, scRNA-seq

## Abstract

**Background:**

The complement system expressed intracellularly and known as complosome has been indicated as a trigger in the regulation of lymphocyte functioning. The expression of its genes was confirmed also in several types of human bone marrow-derived stem cells: mononuclear cells (MNCs), very small embryonic-like stem cells (VSELs), hematopoietic stem/progenitor cells (HSPCs), endothelial progenitors (EPCs) and mesenchymal stem cells (MSCs). In our previous studies, we demonstrated the expression of complosome proteins including C3, C5, C3aR, and cathepsin L in purified HSPCs. However, there is still a lack of results showing the expression of complosome system elements and other immunity-related proteins in human HSPCs at the level of single cell resolution.

**Methods:**

We employed scRNA-seq to investigate comprehensively the expression of genes connected with immunity, in two populations of human HSPCs: CD34+Lin-CD45+ and CD133+Lin-CD45+, with the division to subpopulations. We focused on genes coding complosome elements, selected cytokines, and genes related to antigen presentation as well as related to immune regulation.

**Results:**

We observed the differences in the expression of several genes e.g. C3AR1 and C5AR1 between two populations of HSPCs: CD34+LinCD45+ and CD133+Lin-CD45+ resulting from their heterogeneous nature. However, in both kinds of HSPCs, we observed similar cell subpopulations expressing genes (e.g. NLRP3 and IL-1β) at the same level, which suggests the presence of cells performing similar functions connected with the activation of inflammatory processes contributing to the body's defense against infections.

**Discussion:**

To our best knowledge, it is the first time that expression of complosome elements was studied in HSPCs at the single cell resolution with the use of single cell sequencing. Thus, our data sheds new light on complosome as a novel regulator of hematopoiesis that involves intracrine activation of the C5a-C5aR-Nlrp3 inflammasome axis.

## 1 Introduction

Hematopoiesis and the immune system originate from a common hematopoietic/lymphopoietic stem cell. This close developmental relationship explains that cells belonging to both lineages often share the same receptors and respond to similar factors (1–7). Gene expression analysis performed on bulk RNA supported this developmental origin, explained by the common goal of both lineages being involved in tissue homeostasis, fighting invading pathogens, and promoting tissue repair (8–12). The complement, a crucial innate and adaptive immunity element, is a complex multiprotein system responsible for inflammatory processes and other biological effects (13–16). Initially, it was considered a serum-effective system in which proteins were synthesized in the liver and released into circulation (17, 18). Recent data demonstrated the presence of complement elements expressed intracellularly, which is crucial for the normal functioning of lymphocytes (19–21). Furthermore, the expression of its components was also detected in several types of human bone marrow-derived stem cells, including mononuclear cells (MNCs), very small embryonic-like stem cells (VSELs), hematopoietic stem/progenitor cells (HSPCs), endothelial progenitors (EPCs) and mesenchymal stem cells (MSCs) (22). Our previous studies demonstrated the expression of functional complosome proteins, including C3, C5, C3aR, and cathepsin L in purified human and murine HSPCs (18, 22). It has also been reported that HSPCs express several receptors that protect them from potential damage by circulating in peripheral blood (PB) activated complement proteins (23), some members of the toll-like receptor family (TLRs) (24, 25), and intracellular pattern recognition receptors (PRRs) (26, 27). Pattern recognition receptors (PRRs) are germline-encoded sensors that detect pathogen-associated (PAMPs) or damage-associated molecular patterns (DAMPs). They are highly expressed in innate immune cells, such as dendritic cells, macrophages, monocytes, and epithelial cells Some elements of innate immunity, such as intracellular PRR, have been implicated, for example, in the specification of HSPCs in mammals during embryogenesis (28) and being involved in the early stages of hematopoiesis in the zebrafish (29, 30). Furthermore, bone marrow (BM) cells from TLR4−/−, TLR9−/−, and MyD88−/− mice show a repopulating advantage over wild-type cells in lethally irradiated recipients (28), suggesting that TLR signaling supports HSPC maintenance.

To our best knowledge, there is still a lack of information about the expression and functioning of the complosome elements, obtained from single-cell sequencing experiments. This method (scRNA-seq) is becoming an increasingly widely used tool in biological and biomedical research and has advanced the understanding of a range of biological processes and molecular mechanisms occurring in cells. It potentially may have a clinical application and may be valuable in new diagnostic and therapeutic strategies (31). In the case of hematopoietic stem cells, scRNA-seq allowed to decode both human (32) and mice (33) hematopoiesis. Furthermore, the first hematopoietic stem cells in human embryo were traced by scRNA-seq (34). In our previous scRNA-seq experiment (35), we identified several HSPCs subpopulations (14 clusters for both CD34+Lin-CD45+ and CD133+Lin-CD45+, with their basic division into 2 main cell groups. One, with confirmed expression of expression of two stem cell markers: CD34 and PROM1 (CD133). We call them primitive or quiescent clusters. And another one, with confirmed expression of lymphocyte (T and B), monocytes, macrophages, NK cells and granulocytes markers (e. g. CD2, CD3, CD19, CD14, FCGR3A, CD68). We call them ‘fate decision’ clusters (35). In the current study, we employed scRNA-seq and untargeted proteomic analysis using mass spectrometry to investigate the expression of genes immunity in two populations of purified human umbilical cord blood (UCB) HSPCs: CD34+Lin-CD45+ and CD133+Lin-CD45+, paying special attention to track the differences between primitive and ‘fate decision’ clusters. We analyzed our mRNA expression data sets employing the Uniform Manifold Approximation and Projection for Dimension Reduction (UMAP) technique, which allows us to visualize separate cell clusters (36, 37). We focused on genes encoding proteins involved in immune responses, encoding complosome elements, and selecting cytokines. To our knowledge, it is the first time that the expression of all these genes was studied in HSPCs at the single-cell resolution using single-cell sequencing. We observed some similarities and differences in gene expression between CD34+Lin-CD45+ and CD133+Lin-CD45+ cells. Moreover, scRNA-seq results revealed significant differences from previously published data using bulk mRNA analyses. Finally, the expression of some of these genes was subsequently evaluated by proteomic analysis.

## 2 Materials and methods

### 2.1 Isolation of human CD34+ and CD133+ HSPCs

Human umbilical cord blood (hUCB) was obtained from a healthy newborn delivered at the Department of Obstetrics and Gynecology, Medical University of Warsaw (Warsaw Bioethics Committee permission number KB/50/2022). Blood was diluted with phosphate-buffered saline (PBS) and carefully layered over Ficoll-Paque (GE Healthcare, Chicago, IL, USA). Next, it was centrifuged for 30 min at 400x g at 4°C. The mononuclear cells (MNCs) were collected, washed, and stained with the following antibodies: hematopoietic lineage markers (Lin) cocktail of antibodies, each FITC-conjugated: CD235a (clone GA-R2 [HIR2]), anti-CD2 (clone RPA-2.10), anti-CD3 (clone UCHT1), anti-CD14 (clone M5E2), anti-CD16 (clone 3G8), anti-CD19 (clone HIB19), anti-CD24 (clone ML5), anti-CD56 (clone NCAM16.2) and anti-CD66b (clone G10F5) (all BD Biosciences, San Jose, CA, USA); PE-Cy7-conjugated anti-CD45 (clone HI30, BioLegend, San Diego, CA, USA), PE-conjugated anti-CD34 (clone 581, BioLegend, San Diego, CA, USA) and APC-conjugated anti-CD133 (clone CD133, MiltenyiBiotec, Gladbach, Germany). Cells were then stained in the dark at 4°C for 30 min. After incubation, they were centrifugated and resuspended in RPMI-1640 medium containing 2% fetal bovine serum (FBS, Corning Inc, Corning, NY, USA). Finally, cells were sorted according to the strategy described in numerous reports (38–40). Briefly, small events (2–15 μm in size) were included in the “lymphocyte-like” gate (P1) and then further analyzed for the expression of the Lin marker. Lin negative events were gated and subsequently analyzed for the expression of CD45 and CD34 or CD133 antigens. Populations of CD34+ HSPCs (CD34+Lin−CD45+) and CD133+ HSPCs (CD133+Lin−CD45+) were obtained on the MoFlo Astrios EQ cell sorter (Beckman Coulter, Brea, CA, USA).

### 2.2 Single-cell sequencing

After sorting, cells were directly proceeded using Chromium X Controller (10X Genomics, USA) and Chromium Next GEM Chip G Single Cell Kit (10X Genomics, USA). Chromium Next GEM Single Cell 3’ GEM, Library & Gel Bead Kit v3.1, and Single Index Kit T Set A (10X Genomics, USA) were used for library preparation according to manufacturer’s guidelines. Libraries were then pooled and run on Illumina NextSeq 1000/2000 (Illumina, San Diego, CA, USA) in P2 flow cell chemistry (200 cycles) with paired-end sequencing mode (read 1–28 bp, read 2–90 bp, index 1 – 10 cycles, index 2 – 10 cycles), assuming 25,000 reads per single cell.

### 2.3 Bioinformatic analysis

Downstream analysis was performed using Seurat (version 5.0.1), preceded by the 10X Genomics Cell Ranger pipelines (CellRanger version 7.2.0, 10x Genomics, USA) (41, 42). Raw sequencing files (BCL files) were demultiplexed and converted to fastq files using the bcl2fastq (version v2.20.0.422) within the 10X Genomics Cell Ranger mkfastq pipeline (43, 44). Then, the Cell Ranger count pipeline was used. Cells with less than 200 and more than 2500 transcripts and those with more than 5% of mitochondria-related transcripts were excluded from the analysis. Sequencing reads were mapped to a human genome GRCh38 (version 2020-A) acquired from the 10×Genomics website (https://www.10xgenomics.com/support/software/cell-ranger/downloads#reference-downloads). Gene expression measurements for each cell were normalized, and the normalized values were log-transformed (“LogNormalize” method) and then reduced to the first 2000 most highly variable genes. Non-linear dimensional reduction to visualize clusters was performed using uniform manifold approximation and d projection (UMAP) implemented in Seurat (version 5.0.1.). Cell clusters were recognized based on differentially expressed genes, both positive (up-regulated genes) and negative (down-regulated genes) markers, using an adjusted p-value <0.05 and a log2FC >1. However, for this publication, we focused only on positive ones.

### 2.4 Data visualization

Data visualization was done using R (R Core Team) with the ggplot2 R package (version 3.4.4) (45). We selected hematopoietic lineage differentiation cell markers (CD2, CD3, CD4, CD11b (ITGAM), CD14, CD16 (FCGR3A), CD19, CD34, CD41 (ITGA2B), CD45 (PTPRC), CD66b (CEACAM8), CD68, GYPA, PROM1 (CD133), CD117 (c-KIT) to determine their expression in CD34+lin-CD45+ and CD133+lin-CD45+ and confirm the hematopoietic character of the studied cells. Furthermore, we selected several genes related to the complosome system and immunity. They were as follows: TLR1, TLR2, TLR3, TLR4, TLR5, TLR6, TLR7, TLR8, TLR9, TLR10, NOD1, NOD2, NOD3, NOD4, NOD5, NLRC4, CIITA, NAIP, NLRP1, NLRP2, NLRP3, NLRP4, NLRP5, NLRP6, NLRP7, NLRP8, NLRP9, NLRP10, NLRP11, NLRP12, NLRP13, NLRP14, C1QA, C1QB, C1QC, C1R, C1S, C2, C3, C4, C5, C6, C7, C8, C9, CFB, CFP, C3AR1, C5AR1, C5AR2, CTSL, CFH, CFI, CD46, CD55, CD59, C1NH, CFHR1, CFHR2, CFHR3, CFHR4, CFHR5, CASP1, IL1B, IL18, PYCARD, MEFV, AIM2, MDAS, DDX58.

### 2.5 Data availability

The data sets supporting this article’s results are available in the Sequence Read Archive (SRA) repository (https://www.ncbi.nlm.nih.gov/sra) and assigned a unique persistent identifier: PRJNA1128409 (https://www.ncbi.nlm.nih.gov/sra/PRJNA1128409).

### 2.6 Mass spectrometry

MoFlow-isolated CD133+lin-CD45+ and CD34+lin-CD45+ HSPCs were lysed in 50 μL of RIPA buffer. Samples were incubated for 60 min at 96°C and then sonicated for 90 min in a water bath. Samples were precipitated with the use of ice cold (−20°C) Acetonitrile (ACN, Merck, Darmstadt, Germany) in a volume 1:4 ratio and incubated for 120 min in (−20°C). After precipitation, samples were centrifuged (30 min, −9°C, 18000 × g), and the supernatant was removed. The protein pellet was dissolved in 40 mM ammonium bicarbonate (Merck, Darmstadt, Germany). Reduction and alkylation were performed using 500 mM DTT (in a final concentration of 20 mM) and 1 M iodoacetamide (IAA, in a final concentration of 40 mM). Proteins were in-solution digested for 16 h at 37°C using Trypsin Gold (Promega, Madison, WI, United States).

Liquid chromatography-mass spectrometry (LC-MS) analysis was carried out with the use of nano-UHPLC (nanoElute, Bruker, Billerica, MA, United States) coupled by Captive Spray (Bruker, Billerica, MA, United States) to ESI-Q-TOF mass spectrometer (Compact, Bruker, Billerica, MA, United States). A two-column separation method was used, that is, pre-column (300 μm × 5 mm, C18 PepMap 100, 5 μm, 100 Å, Thermo Scientific, Waltham, MA, United States) and Bruker fifteen separation column (75 μm × 150 mm, C18 1.6 μm) in gradient 2% B to 55% B in 45 min with the 300 nL/min flow rate.

### 2.7 Proteomic data analysis

The collected spectra were calibrated (lock mass calibrant) and analyzed using Data Analysis software (Bruker). They were then identified in ProteinScape (Bruker) using the MASCOT server. Proteins were identified using the online SwissProt database, and their annotation and biological significance were determined using UniProt.org and KEGG. All analyses were performed in the R version [4.3.3]. The following R packages were employed for data processing, statistical analysis, and visualization: tidyverse (46) for data manipulation and wrangling, readxl (47) for importing Excel files, dplyr (48) and purrr (49) for functional programming and data cleaning, and writexl (50) for exporting processed results to Excel.

## 3 Results

### 3.1 Cell subpopulations within CD34+Lin-CD45+ and CD133+Lin-CD45+

**Figure 1:**
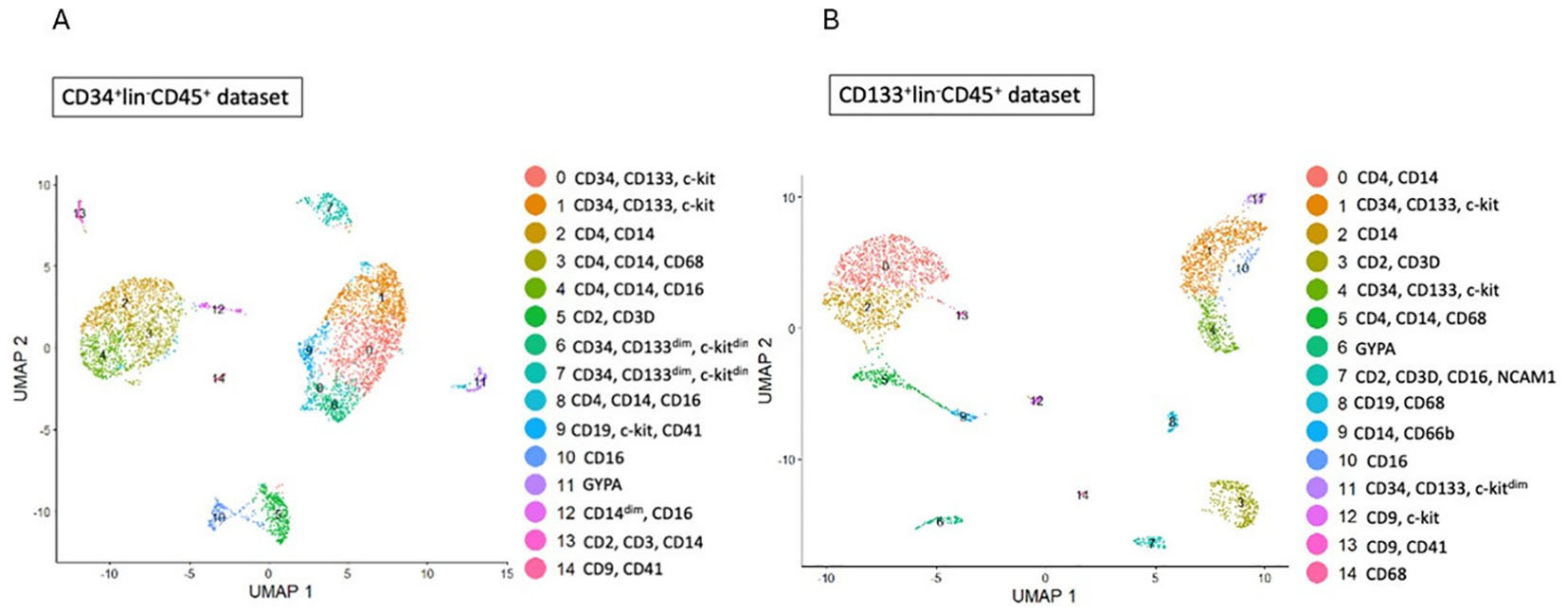
Identification of subpopulations in studied samples of HSCs. **(A, B)** - CD34+lin-CD45+ **(A)**, CD133+lin-CD45+ **(B)** visualized by UMAP method. The datasets are first analysed without integration. The resulting clusters are defined both by cell type Uniform manifold approximation and projection (UMAP) plot.

We identified several cell types and cell subpopulations in CD34+Lin-CD45+ (**Figure 1A**) and CD133+Lin-CD45+ (**Figure 1B**) cells, as it was previously published (35). Cluster 0 and 1 of CD34+Lin-CD45+ cells and clusters 1 and 4 of CD133+Lin-CD45+ cells were identified to express transcripts of CD34, CD133 (PROM1), and c-kit genes, corresponding to the most primitive cells evaluated in our study. Additionally, we also identified clusters 6 and 7 of CD34+Lin-CD45+ (**Figure 1A**) as cells expressing CD34, CD133^dim^, and c-kit^dim^ and cluster 11 of CD133+Lin-CD45+ (**Figure 1B**) as primitive cells expressing CD34, CD133 and c-kit^dim^. Furthermore, both HSCPs populations (**Figures 1A, B**) contain cell clusters with confirmed expression of myeloid (CD14, CD68) and lymphoid (CD2, CD4) progenitors. We call them “fate decision” clusters.

### 3.2 Expression of toll-like receptors

**Figure 2:**
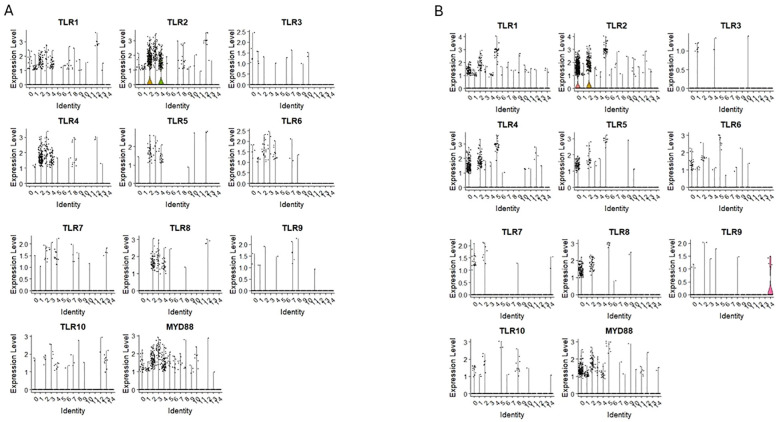
The expression of Toll-like receptor (TLRs) genes in CD34+lin-CD45+ **(A)** and CD133+lin-CD45+ **(B)** cells with the division to clusters. Violin plots shows differential expression of TLR1, TLR2, TLR3, TLR4, TLR5, TLR6, TLR7, TLR8, TLR9, TLR10 and MYD88 in different subpopulations of both datasets.

Our scRNA-seq data (**Figure 2**) revealed the expression of transcripts of TLR1 and TLR 2 receptors in primitive clusters (0 and 1 of CD34+lin-CD45+ and 1 of CD133+lin-CD45+), as well as fate decision clusters (2, 3 and 4 of CD34+lin-CD45+; and 0, 2 and 5 of CD133+lin-CD45+) Additionally, TLR4, TLR5, TLR6, and TLR8, were also expressed on cells in clusters enriched of CD2, CD4, CD14 and CD68 mRNA in both datasets (**Figures 2A, B**).

### 3.3 Expression of NOD-like receptors

Next, we evaluated mRNA expression for NOD-like receptors in our scRNA-seq data sets:

-CARD domain – containing NODs/NLRCs

**Figure 3:**
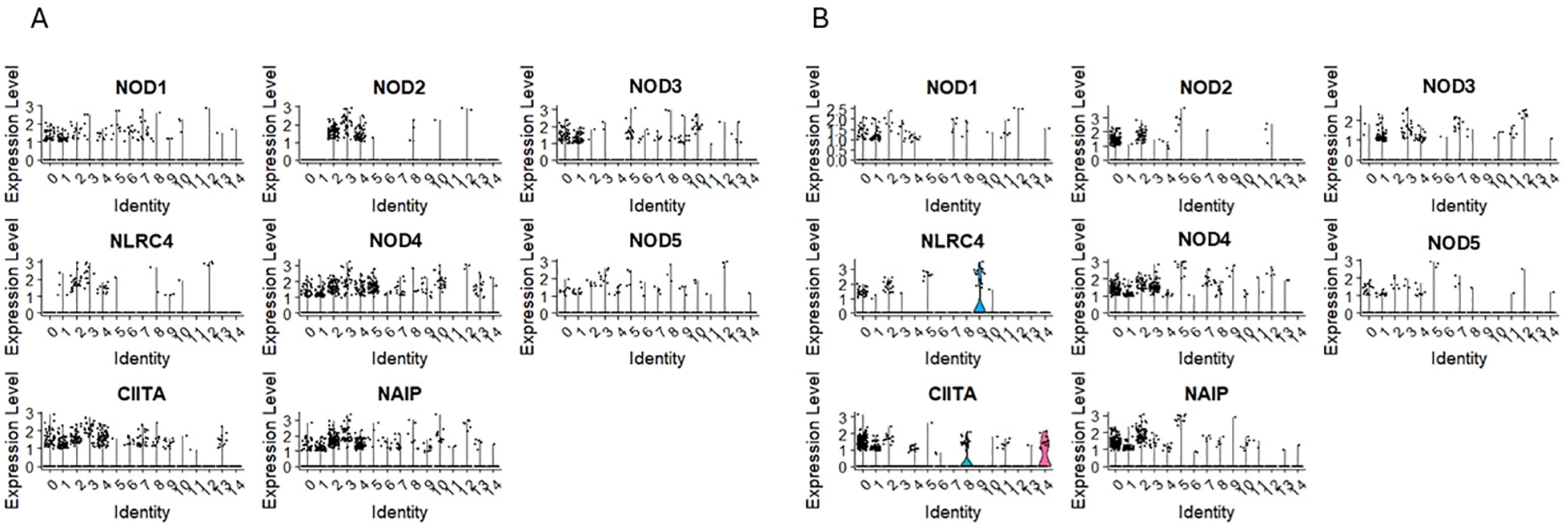
The expression of CARD domain–containing NOD-like receptor (NOD) genes in CD34+lin-CD45+ **(A)** and CD133+lin-CD45+ **(B)** cells with the division to clusters. Violin plots show differential expression of NOD1, NOD2, NOD3, NLRC4, NOD4, NOD5, CIITA, and NAIP in different subpopulations of both datasets.

The nucleotide-binding oligomerization domain-like receptors, or NOD-like receptors (NLRs), are intracellular sensors known as pattern recognition receptors (51, 52). We detected transcripts for NOD1/NLRC1, NOD3/NLRC3, NOD4/NLRC5, NOD5/NLRZ1, CIITA/NLRA and NAIP in clusters 0 and 1 for CD34+Lin-CD45+ (**Figure 3A**) and clusters 1 and 4 for CD133+Lin-CD45+ (**Figure 3B**) enriched for mRNA for CD34, CD133, and c-kit (primitive clusters). The mRNA for these receptors was also expressed in other cell clusters with confirmed expression of mRNA for lymphocytic precursors.

-Pyrin domain – containing NODs/NLRPs

**Figure 4:**
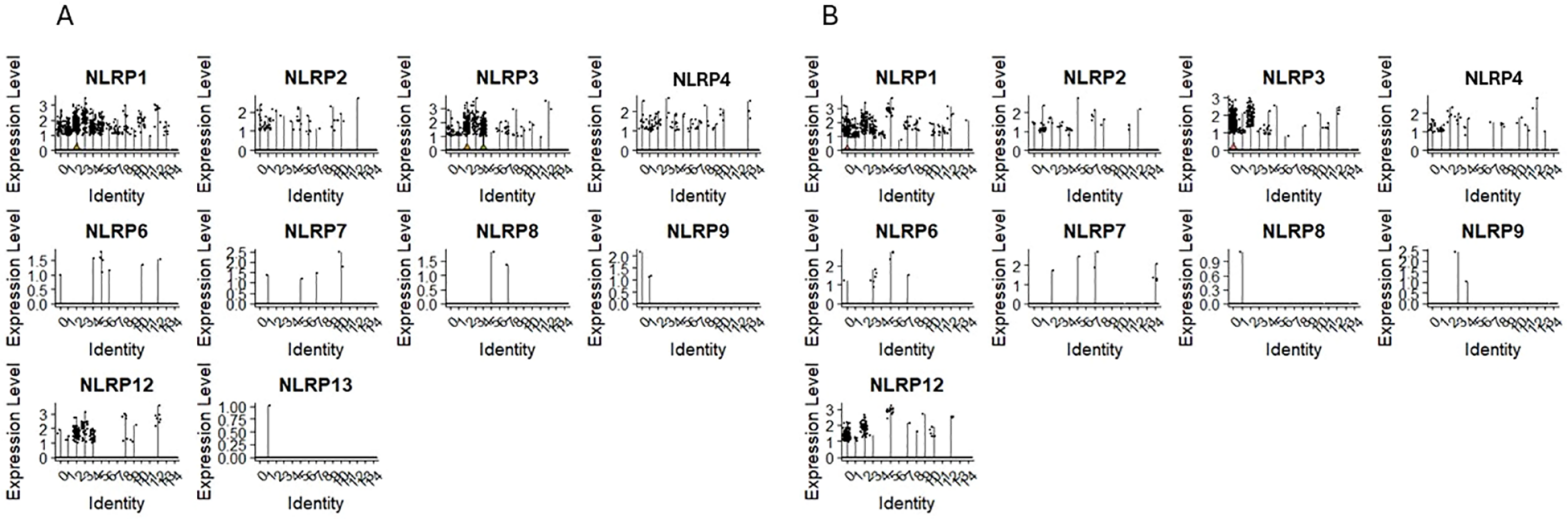
The expression of Pyrin domain–containing NOD-like receptor (NOD) genes in CD34+lin-CD45+ **(A)** and CD133+lin-CD45+ **(B)** cells with the division to clusters. Violin plots shows differential expression of NLRP1, NLRP2, NLRP3, NLRP4, NLRP6, NLRP7, NLRP8, NLRP9, NLRP12 and NLRP13 in different subpopulations of both datasets.

The expression of NLRP1, NLRP2, NLRP3, NLRP4/PAN2 was observed in clusters 0 and 1 of CD34+Lin-CD45+ (**Figure 4A**) and clusters 1 and 4 of CD133+Lin-CD45+ (**Figure 4B**), which are, as it was mentioned above the most primitive cell subpopulations of HPSCs (expressing CD34, CD133, and c-kit transcripts). Additionally, their expression was observed in “fate decision” subpopulations, with confirmed expression of CD4 and CD14 in both HSPCs populations. The same was found for NLRP12, which was also expressed in cell clusters expressing mRNA for lymphocytic precursors (CD4, CD14, CD68). Expression of NLRP5, NLRP10, NLRP11, and NLRP14 was not observed in both scRNA-seq datasets.

### 3.4 Expression of mRNA for RIG-1-like receptors and AIM2

**Figure 5:**
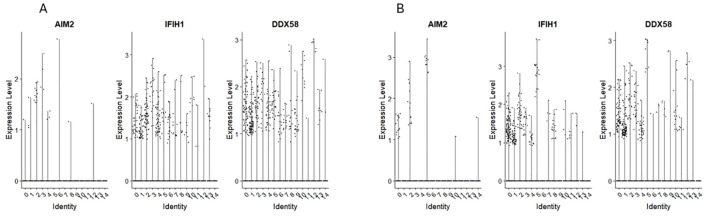
The expression of RIG-like receptors genes in CD34+lin-CD45+ **(A)** and CD133+lin-CD45+ **(B)** cells with the division to clusters. Violin plots show differential expression of AIM2, IFIH1, and RIG-1 in different subpopulations of both datasets.

We found the expression of RIG-1-like receptors MDAS (IFIHI) and DDX58 in clusters 0 and 1 of CD34+Lin-CD45+ (**Figure 5A**) and clusters 1 and 4 of CD133+Lin-CD45+ (**Figure 5B**).

AIM2 expression was found however, its level was very slight and observed only in single cells of both HSPCs populations.

### 3.5 Expression of mRNA for complement cascade/complosome

**Figure 6:**
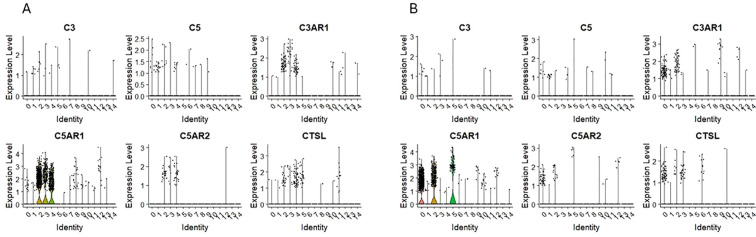
The expression of complement system genes in CD34+lin-CD45+ **(A)** and CD133+lin-CD45+ **(B)** cells with the division to clusters. Violin plots show differential expression of C3, C5, C3AR1, C5AR1, C5AR2, and CTSL in different subpopulations of both datasets.

We found the expression of C3 and C5 in both HSPCs populations: CD34+lin-CD45+ (**Figure 6A**) and CD133+lin-CD45+ (**Figure 6B**) however it was on the slight level with just a few cells expressing mRNA of these genes. Interestingly, the expression of C3AR1, C5AR1, and C5AR2 (**Figures 6A, B**) was observed not in all cells of both HSPCs populations but in the subpopulations with confirmed expression of CD4 and CD14 markers, corresponding to ‘fate decision’ cells, where the mechanisms of immunological actions start to become active. Because the expression of receptor genes is observed only in this group of cells, we can assume that their later immunological potential comes from there. We do not see the expression of C3, C5, C3AR1, C5AR1, and C5AR2 (**Figures 6A, B**) in the quiescent subpopulation of HSPCs with a confirmed expression of CD34, CD144, and c-KIT. Interestingly, in both HSPCs populations: CD34+lin-CD45+ and CD133+lin-CD45+.

### 3.6 Expression of mRNA for NLRP3 inflammasome components

**Figure 7:**
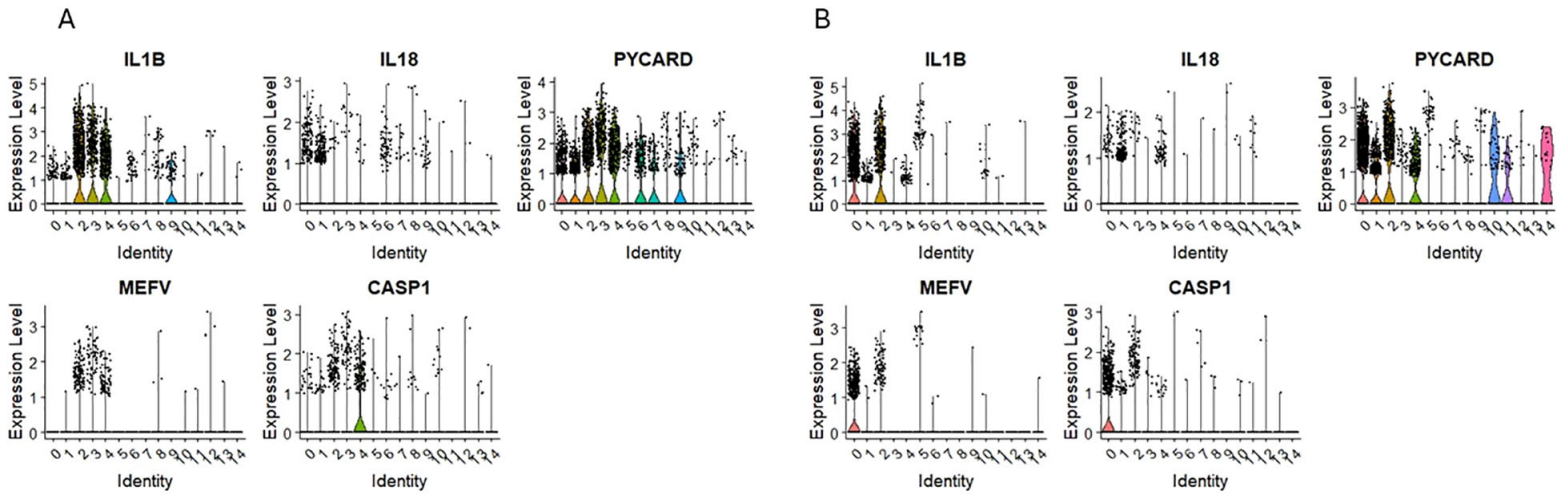
The expression of innate immunity genes in CD34+lin-CD45+ **(A)** and CD133+lin-CD45+ **(B)** cells with the division to clusters. Violin plots show differential expression of IL1B, IL18, PYCARD, MEFV, and CASP1 in different subpopulations of both datasets.

Our scRNA-seq datasets revealed that mRNA for Nlrp3 inflammasome is expressed in clusters 0 and 1 for CD34+Lin-CD45+ and UMAP clusters 1 and 4 for CD133+Lin-CD45+ enriched for mRNA of HSPCs (**Figure 4**). In addition, **Figures 7A, B** show mRNA expression for crucial components of this pattern recognition receptor, including mRNA for IL1β, IL18, PYCARD, and CASP1 in the same clusters. The NOD-like receptor protein 3 (NLRP3) inflammasome is a protein complex that regulates innate immune responses by activating caspase-1 and the inflammatory cytokines interleukin (IL)-1β and IL-18 (53–55). We and others have demonstrated that Nlrp3 inflammasome is expressed in HSPCs and regulates the trafficking and metabolism of these cells (56–59).

### 3.7 Expression of mRNA for complement regulatory proteins

**Figure 8:**
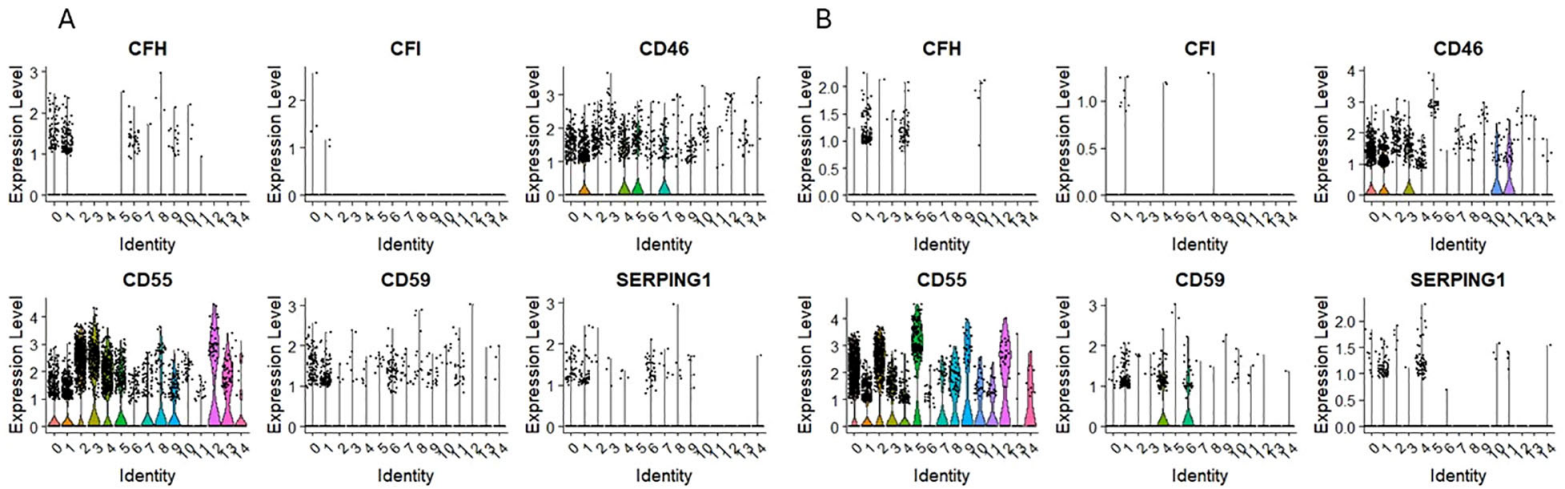
The expression of complement system regulatory genes in CD34+lin-CD45+ **(A)** and CD133+lin-CD45+ **(B)** cells with the division to clusters. Violin plots show differential expression of CFH, CFI, CD46, CD55, CD59, and SERPING1 in different subpopulations of both datasets.

Opposite to complement system genes, which expression was mostly observed in ‘fate decision’ HSPCs subpopulations; the expression of complosome regulatory elements was found in the primitive, quiescent subpopulations in both HSPCs datasets. Complement factor H (CFH) expression was found only in cells expressing CD34, CD133, and c-KIT in CD34+lin-CD45+ and CD133+lin-CD45+ cells (**Figures 8A, B**). Additionally, the expression of CD59 was found primarily on primitive subpopulations (**Figures 8A, B**); however, in the case of CD34+lin-CD45+, it was also found in cells expressing GYPA (**Figure 8B**).

### 3.8 Comparative analysis of proteins identified in HSPCs datasets

**Figure 9:**
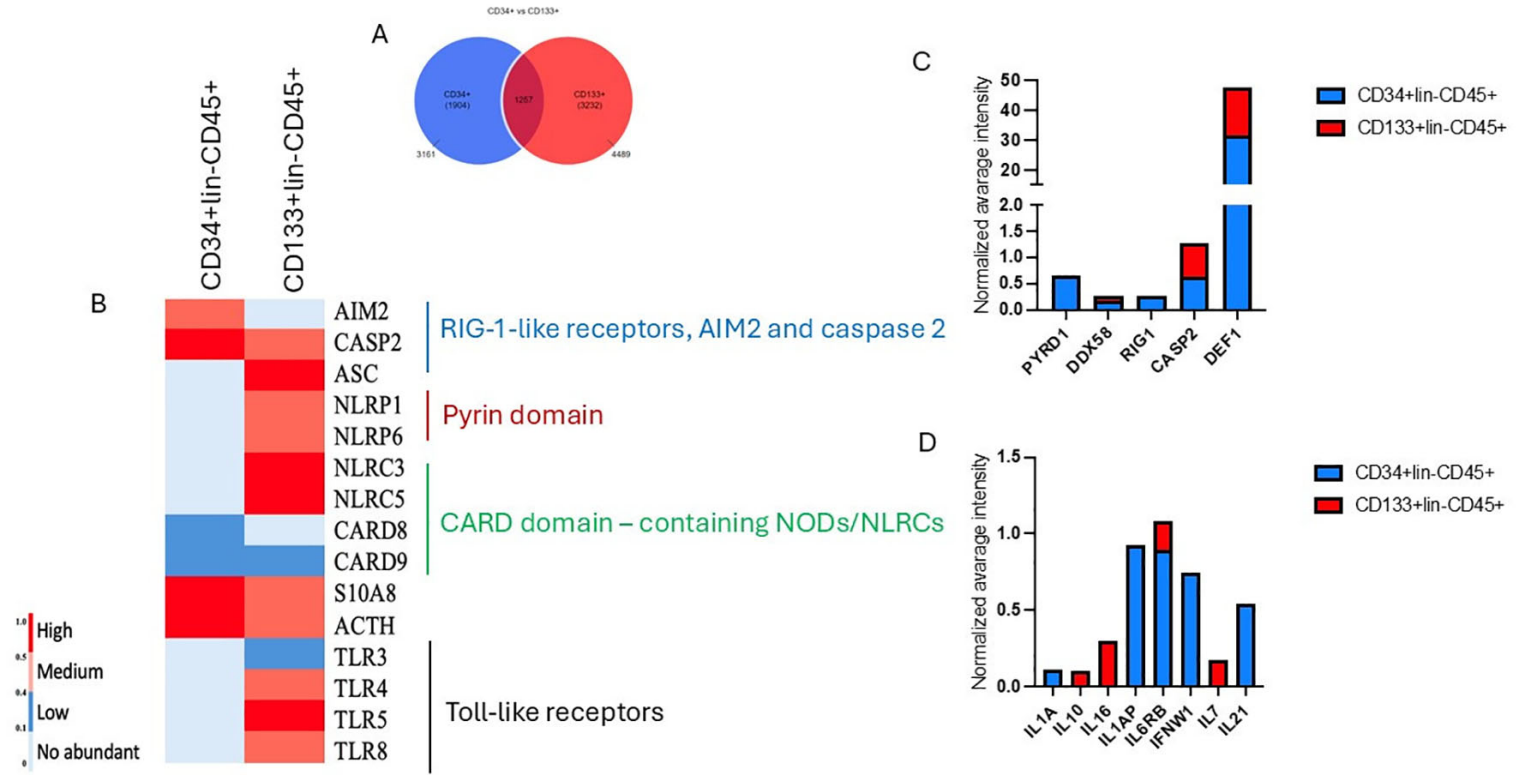
Analysis of annotated proteins to CD34+lin-CD45+ and CD133+lin-CD45+ HSPCs dataset. **(A)** Vienna plot of annotated proteins for both datasets. **(B)** Comparative analysis of the mean normalized heat map for CD34+ and CD133+HSPCs dataset presents annotated common proteins corresponding to GO biological processes regulating immune response, inflammation, and cell death. **(C, D)** The mean normalized intensity displays the abundance of annotated proteins playing a pivotal role in the inflammation.

Using untargeted mass spectrometry for proteomic analysis, we identified 3161 and 4489 proteins for CD34+ and CD133+ HSPCs datasets (**Figure 9A**). Among those, 1257 proteins were common for both datasets. The highly enriched GO biological pathways and the number of proteins were involved in innate and adaptive immunity and inflammation (**Figure 9B**). Heatmap comparison of mean normalized intensities (**Figure 9B**) shows the comparative analysis of Toll-like receptors (TLR3, TLR4, TLR5) more abundant in CD133+lin-CD45+ dataset, proteins with pyrin (PYD) domain, that are most recognized for its role in signaling innate immunological responses, specifically in the formation of large protein complexes called inflammasomes, which enable the activation of caspase-1 by promoted proximity-mediated activation and the subsequent release of pro-inflammatory cytokines (60). We also detected the presence of AIM2 and ACS in our datasets, however a high abundance of AIM 2 was detected for CD34+lin-CD45+ HSCS, whereas higher mean normalized intensity was observed for ASC CD133+lin-CD45+ HSPCs.

Simultaneously, we detected increased caspase 2 and defensin for the CD133+ HSPCs dataset (**Figure 9C**). We also employed the Uniprot database and identified several immunomodulatory cytokines, including IL1, IFN, IL10, IL21 and IL-7 (**Figure 9D**).

## 4 Discussion

Using scRNA-seq, we confirmed that mRNA for some receptor and protein components of the immune system are expressed in purified human HSPCs, indicating cell subpopulations in which these genes are up or down regulated. There are published studies in which mRNA isolated as bulk from cells enriched for HSPCs were employed for RT-PCR analysis (18, 22, 61). To address this issue better, we used prospectively isolated by FASCs human umbilical cord blood (UCB)-derived CD34+Lin-CD45+ and CD133+Lin-CD45+ cells and performed single-cell RNA-seq (scRNA-seq) analysis to detect mRNA of genes involved in immune responses. We analysed our mRNA expression data sets employing the Uniform Manifold Approximation and Projection for Dimension Reduction (UMAP) technique, which allows us to visualize separate cell clusters (36). Since both CD34+Lin-CD45+ and CD133+Lin-CD45+ cell populations are enriched for the cells at different levels of specification toward hematopoietic and lymphoid lineages, it was challenging to evaluate the expression of innate immunity markers in these cells. In particular, we were interested in UMAP clusters enriched for mRNA encoding antigens CD34, CD133, and c-kit receptors. We also employed untargeted mass spectrometry for proteomic analysis and identified several biological pathways and the number of proteins involved in innate and adaptive immunity and inflammation.

First, we evaluated the expression of membrane-bound and intracellular pattern recognition receptors (PPRs). PPRs are germline-encoded host sensors, which detect molecules typical for the pathogens (pathogen-associated molecular patterns – PAMPs) or release from damaged/apoptotic tissues (damage-associated molecular patterns DAMPs) (62–64). They are highly expressed by innate immunity cells, including dendritic cells, macrophages, monocytes, and epithelial cells (64). Interestingly, as reported, BM cells from TLR4−/−, TLR9−/−, and MyD88−/−mice have a repopulating advantage when transplanted competitively with wild-type marrow into lethally irradiated recipient animals (27, 65, 66). This suggests that TLR signaling may contribute to the maintenance of HSPCs under homeostatic conditions and that endogenous TLR ligands, such as oligosaccharide (LPS) produced by normal gut flora, may regulate HSPCs under steady-state conditions after interacting with selected TLRs (67). By employing scRNA-seq, we identified that cells enriched for mRNA encoding CD34, CD133, and c-kit express also TLR1, TLR2, and TLR6. We also detected mRNA for MYD88, which plays an important role in intracellular signalling from these receptors. Humans lack TLR11, TLR12, and TLR13 in contrast to rodents (62). The expression of Toll-like receptors is corroborated by mRNA expression for MYD88, an adaptor protein required for transducing signals from the activated Toll-like receptors (68). The family of Toll-like receptors is a cell surface-expressed protein class that plays a key role in innate immune system responses. They are expressed on sentinel cells, including macrophages and dendritic cells, which recognize structurally conserved molecules derived from microbes (69). It has been postulated that some of them are also expressed in HSPCs (24). The receptors TLR1, TLR2, TLR4, TLR5, TLR6, and TLR10 are located on the cell membrane, whereas TLR3, TLR7, TLR8, and TLR9 are located in cytosol (70, 71). In one of the reports, it has been postulated that human UCB CD34+ cells express TLR2 and TLR4 and are involved in the specification of CD34+ cells into megakaryopoiesis/thrombopoiesis (72). In another report, it was demonstrated that human HSPCs express TLR4 and can directly sense pathogens and, in response, produce cytokines that promote the emergency of granulopoiesis (73, 74). It has also been shown that signalling through TLRs 7/8 induces the differentiation of human BM CD34+ cells along the myeloid lineage and, in another report, that PAM3CSK4, a synthetic triacylated lipopeptide known as TLRs 1/2 agonist, instructs human HSPCs to become specified into myeloid lineages (75). However, these studies still need to address which subpopulation of HSPCs are responsible for these effects at which specification level.

Next, we analysed the expression of cytoplasmic PRRs, including NOD-like receptors (NLRs) and RIG-I-like receptors (RLRs) (51, 76). We noticed that primitive clusters of HSPCs express NOD1, NOD3 (NLRC3), NOD4 (NLRC5), NOD5 (NLRX1), NLRA (CIITA), and NLRB1 (NAIP). We also found the expression in these cells of mRNA for RIG-I-like receptors MDA5/(IFIH1) and DDX58. RIG-1-like receptors are critical sensors of virus infection, mediating the transcriptional induction of type I interferons and other genes that collectively establish an antiviral host response (76, 77). Expression of these receptors was recently demonstrated during the specification of murine embryonic cells into haematopoiesis (78) and hematopoietic precursors in zebrafish (78). To support this in human embryogenesis, NOD1, as part of the RAC1-NOD1-RIPK2-NF-kB axis, is a critical intrinsic inductor that primes endothelial cells before hemogenic endothelial fate switch and HSPCs specification (79). The next member of this family, NOD3 (Nlrc3), is highly expressed in hematopoietic differentiation stages *in vivo* and *in vitro* and is required in hematopoiesis in zebrafish (80). Mechanistically, NOD3 activates the Notch pathway and is indispensable in hematopoietic stem and progenitor cell emergence and expansion of HSPCs (80). Similarly, it has been reported that RIG-I-like receptors are also involved in the specification of HSPCs in the zebrafish model (81).

Interferon-inducible protein AIM2, also known as absent in melanoma 2, is a protein that in humans is a cytoplasmic sensor found in hematopoietic cells that recognizes the presence of double-stranded DNA (dsDNA) of microbial or host cellular origin (82). In our study, AIM2 expression was found however, its level was very slight and observed only in single cells of both HSPCs populations.

The analysis of another group of NOD-like receptors known as NLRPs (83) revealed expression of NLRP1, NLRP2, NLRP3, NLRP4 (PAN2) in human UCB-purified HSPCs expressing mRNA for CD34, CD133, and c-kit. While NLRP1 controls hematopoietic reconstitution after transplantation and NLRP1-KO in the bone marrow microenvironment could significantly relieve bone marrow inflammatory response and promote hematopoietic reconstitution (84), NLRP2 has a similar role (85). In contrast, NLRP3 has been demonstrated to be expressed in human HSPCs and plays an important role in the migration of these cells and in regulating cell metabolism and survival (86). Moreover, NLRP3 inflammasome is activated in aged HSPCs due to mitochondrial stress and SIRT2 inactivation, contributing to the functional decline of these cells during aging (58).

Next, based on exciting data that some types of cells, including lymphocytes, may express complement proteins (complosome) that may be functional inside cells (17, 20, 21), we evaluated the expression of mRNA for major components of the complement cascade in our purified from UCB HSPCs and focused on cell clusters enriched for mRNA for CD34, CD133, and c-kit. We noticed expressions of C5, C3, and C5aR1. Increased expression was observed mainly in the case of C3AR1 and C5AR1 but also C5AR2 in both HSPCs populations. which are receptors of C3a and C5a subunits. C3AR1 receptor, after binding to C3a, can initiate a signaling cascade leading to inflammatory reactions such as mast cell degranulation, cytokine secretion, and leukocyte chemotaxis. Respectively, the C5AR1 receptor, after binding to C5a, one of the most potent inflammatory mediators, can lead to various cellular responses, including increases in blood vessel permeability, neutrophil chemotaxis, and the release of proinflammatory cytokines (87). A low level of expression of C3 and C5 genes with the increased expression level of C3AR1, C5AR1, and C5AR2 genes may indicate a later stage of complosome activation in these cells. Novel data indicates that complement proteins are not synthesized only in the liver. Still, normal lymphocytes also express them and as reported, activate intracellular complement receptors intracrine-dependently (20). This novel regulatory loop operating in lymphocytes has been named complosome (17, 21), and in addition to lymphocytes (20), we have demonstrated it to be expressed in normal HSPCs (22). Some complement protein components expression corroborated our previous studies with bulk mRNA extractions (18, 22). We confirmed Dr. Kemper’s data that complosome is expressed at higher levels in cells committed to lymphocytic lineage (18). Furthermore, we found that it is also expressed in HSPCs, playing an important role in the regulation of their trafficking, metabolism and proliferation (23). Our data with human HSPCs reveal that the more critical component of the complosome is C5. However, since these cells do not express all the components of proximal ComC proteins and C3 activating cathepsin L, other proteases must be involved in the activation/cleavage of C5.

As it was mentioned above, the expression of complosome regulatory elements was found in the primitive, quiescent subpopulations in both HSPCs datasets. Complement factor H (CFH) expression was found only in cells expressing CD34, CD133, and c-KIT in CD34+lin-CD45+ and CD133+lin-CD45+ cells. This regulatory protein controls the activity of the complement system to prevent damage to host tissues. It inhibits the alternative pathway by binding to C3b, accelerating the decay of C3 convertase (C3bBb), and acting as a cofactor for Factor I-mediated cleavage of C3b. Due to this, by differentiating self from non-self, the complement system does not attack its cells. The expression of CFH in quiescent cells confirms their role in preserving inactive cells for further needs. SERPING1 (C1NH) is a gene that codes for a C1 inhibitor. It is crucial in regulating the complement system’s functions, controlling inflammation, and preventing excessive complement activation. We observed its expression mostly in primitive, quiescent subpopulations of HSPCs. Some expression traces were observed in other clusters of both datasets, but their level was very low. CD59, also known as protectin, is a membrane-bound glycoprotein that plays a critical role in regulating the complement system functioning, mainly by inhibiting the formation of the membrane attack complex (MAC). In our study, its expression was found primarily on primitive subpopulations; however, in the case of CD34+lin-CD45+, it was also found in cells expressing GYPA. The complement cascade is tightly regulated to protect host cells, including HSPCs, from indiscriminate attacks by its activated components (13). Complement inhibitors include the plasma serine proteinase inhibitor serpin (C1 inactivator) (88). The classical pathway is also inhibited by surface-bound proteins CD55 (also known as decay accelerating factor or DAF) (89) and CD46 (also known as membrane co-factor protein or MCP) (90). Furthermore, CD59 is an essential regulatory protein of the complement terminal pathway, the membrane attack complex (MAC) (91). In contrast, complement factor H (CFH) is a key regulator of the alternative complement pathway, ensuring that the complement activation is directed against pathogens or harmful materials while preventing damage to host tissue (92). At the same time, complement factor I (CFI), also known as C3b/C4b inactivator, controls complement activation by cleaving C3b and C4b in both cell-bound or fluid phases (93).

Thus, our data on purified human HSPCs analysed based on transcriptome signature and categorized into subpopulations revealed that cells expressing mRNA of CD34, CD133, and c-kit also express several genes characteristic of innate and acquired immunity cells. It is important to realize that even though we sorted cells based on their very early hematopoietic development phenotype characteristic for HSPCs, only a few such cells are clonogenic in *in vitro* functional progenitor assays. These clonogenic cells, however, correspond to UMAP clusters enriched in mRNA for CD34, CD133, and c-kit.

We also employed untargeted mass spectrometry for proteomic analysis and identified several biological pathways and the number of proteins involved in innate and adaptive immunity and inflammation. We observed some similarities and differences between both populations of HSPCs.

For example, Toll-like receptors (TLR3, TLR4, TLR5) and proteins with pyrin (PYD) domain were more abundant in CD133+lin-CD45+ datasets. On the other hand, AIM2 and ACS proteins were detected in both datasets.

## 5 Conclusion

In this study, we provided a comprehensive analysis of human hematopoietic stem and progenitor cells (HSPCs) purified from umbilical cord blood (UCB) using single-cell RNA sequencing (scRNA-seq) and proteomic approaches. Our findings confirm that mRNA for receptors and proteins associated with innate and adaptive immunity are expressed in HSPCs, particularly in subpopulations enriched for CD34, CD133, and c-kit markers. Using scRNA-seq and UMAP clustering, we identified specific patterns of gene expression that shed light on the roles of these immune components in early hematopoietic development.

Notably, we detected the expression of Toll-like receptors (e.g., TLR1, TLR2, and TLR6) and the adaptor protein MYD88, suggesting potential involvement of these receptors in HSPC maintenance and lineage specification. Additionally, cytoplasmic pattern recognition receptors, including NOD-like and RIG-I-like receptors, were expressed in primitive HSPC clusters, implicating these receptors in hematopoietic development and immune response regulation. Furthermore, our proteomic analysis corroborated these findings and revealed differences in the abundance of immune-related proteins between CD34+Lin-CD45+ and CD133+Lin-CD45+ cell populations.

Importantly, we observed the expression of complement components, such as C3AR1, C5AR1, and C5AR2, and regulatory proteins like CFH and SERPING1 in quiescent HSPCs. These findings highlight the potential involvement of intracellular complement signaling (complosome) in preserving HSPC integrity and modulating their response to environmental stimuli.

Collectively, our results provide new insights into the immune properties of human HSPCs, underscoring their potential role in innate and adaptive immune responses. These findings enhance our understanding of HSPC biology and may inform future therapeutic strategies aimed at harnessing the immune capabilities of these cells for regenerative medicine and immune modulation.

## Data Availability

The datasets presented in this study can be found in online repositories. The names of the repository/repositories and accession number(s) can be found below: https://www.ncbi.nlm.nih.gov/genbank/, PRJNA1128409.
